# Supplementary figures and images for: Doublecortin and JIP3 are neural-specific counteracting regulators of dynein-mediated retrograde trafficking
Source: eLife. 2022 Dec 7;11:e82218. doi: 10.7554/eLife.82218 (PMC9799976; doi:10.7554/eLife.82218)

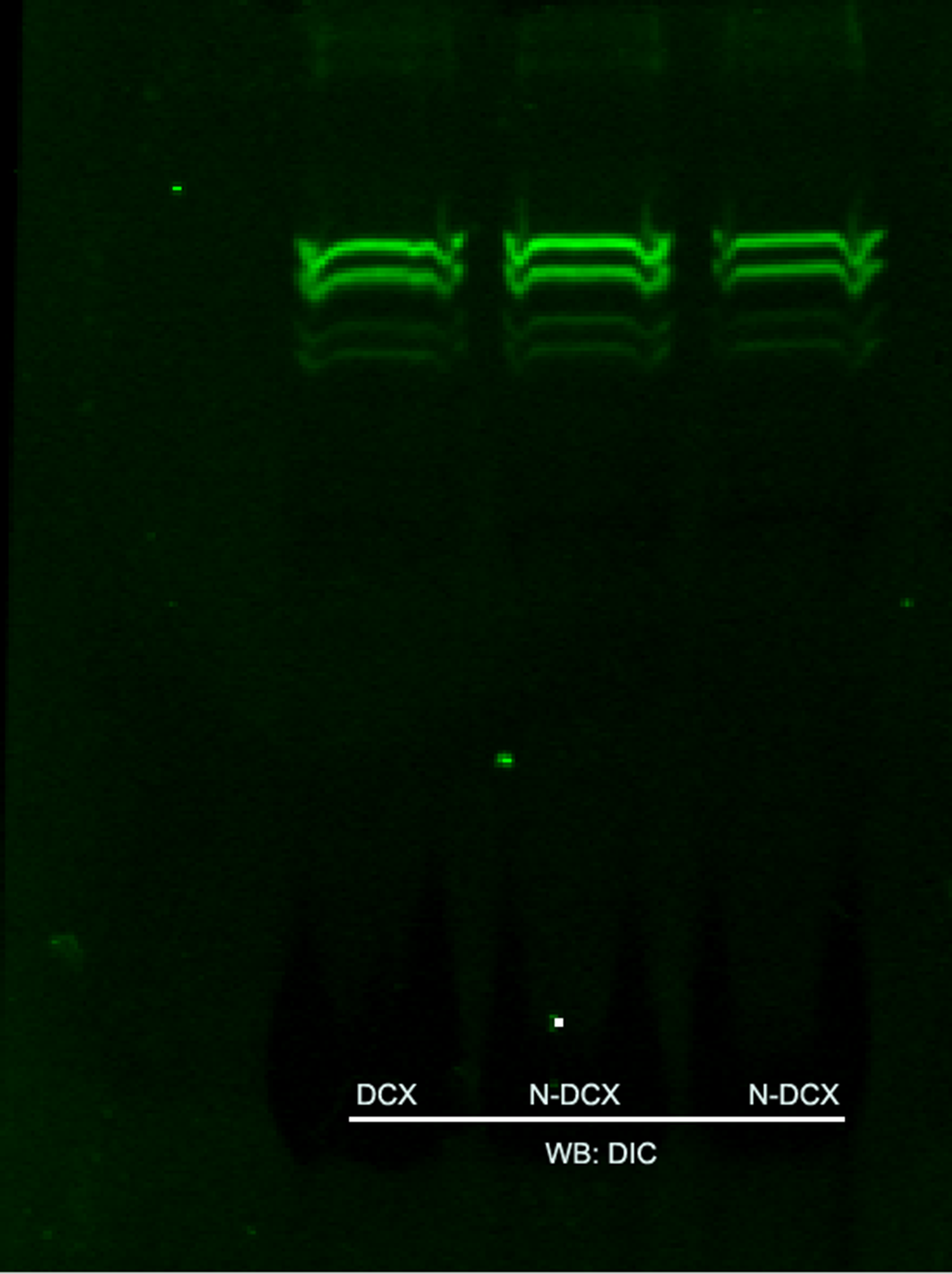

Supplement: Figure 2—source data 1. [file elife-82218-fig2-data1.zip › Fig2B-source data 1.tif]

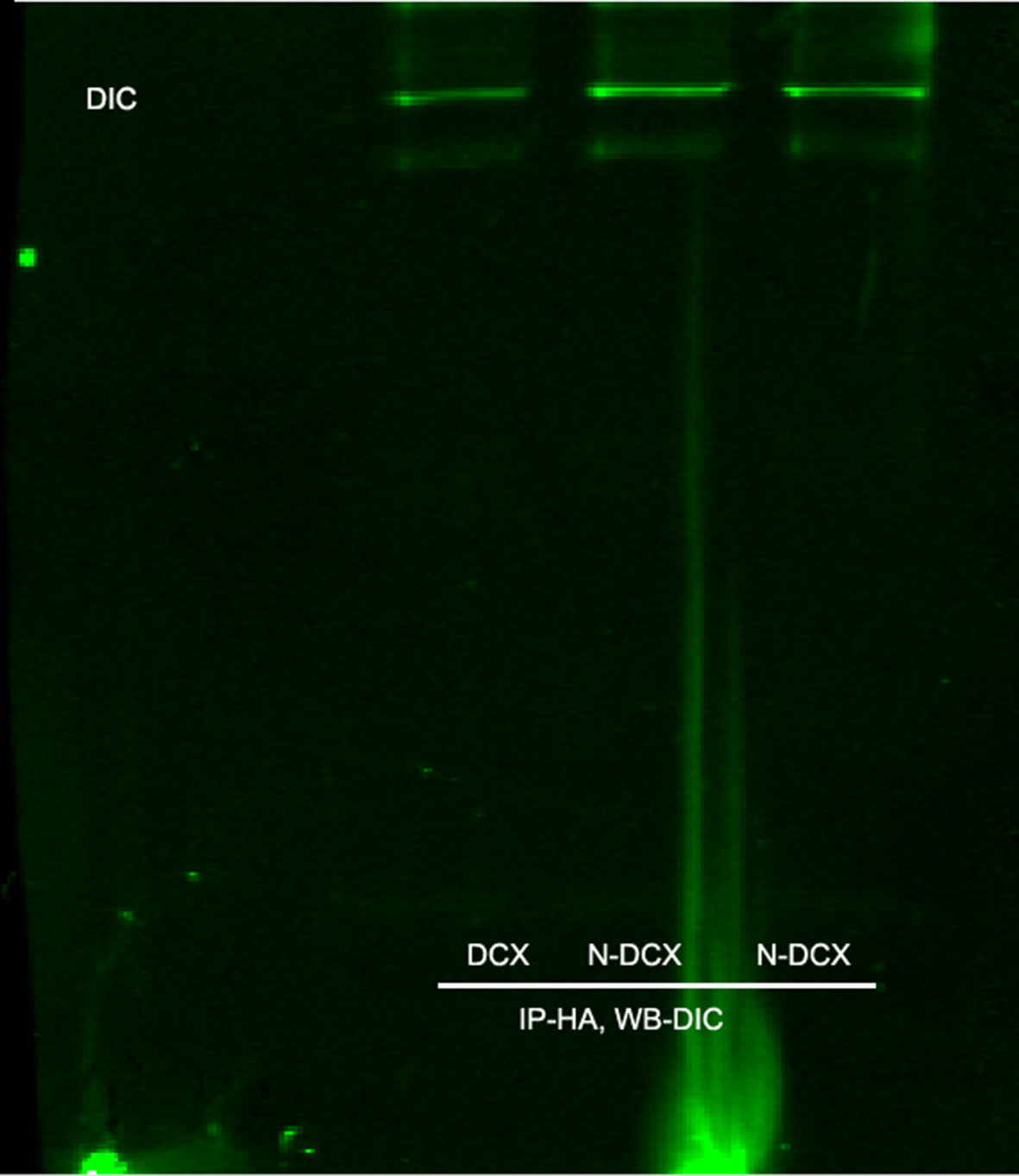

Supplement: Figure 2—source data 1. [file elife-82218-fig2-data1.zip › Fig2B-source data 2.tif]

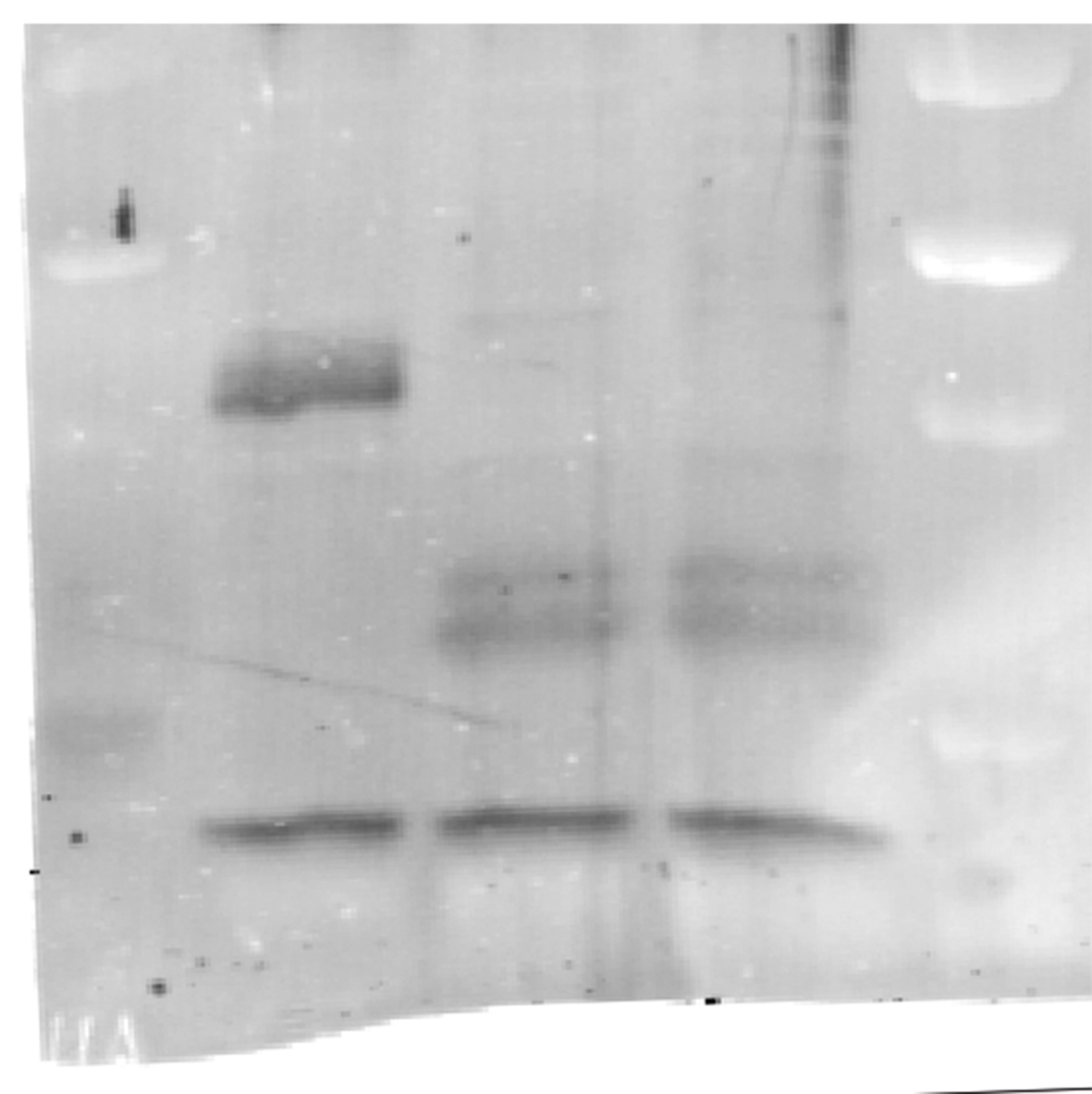

Supplement: Figure 2—source data 1. [file elife-82218-fig2-data1.zip › Fig2B-source data 3.tif]

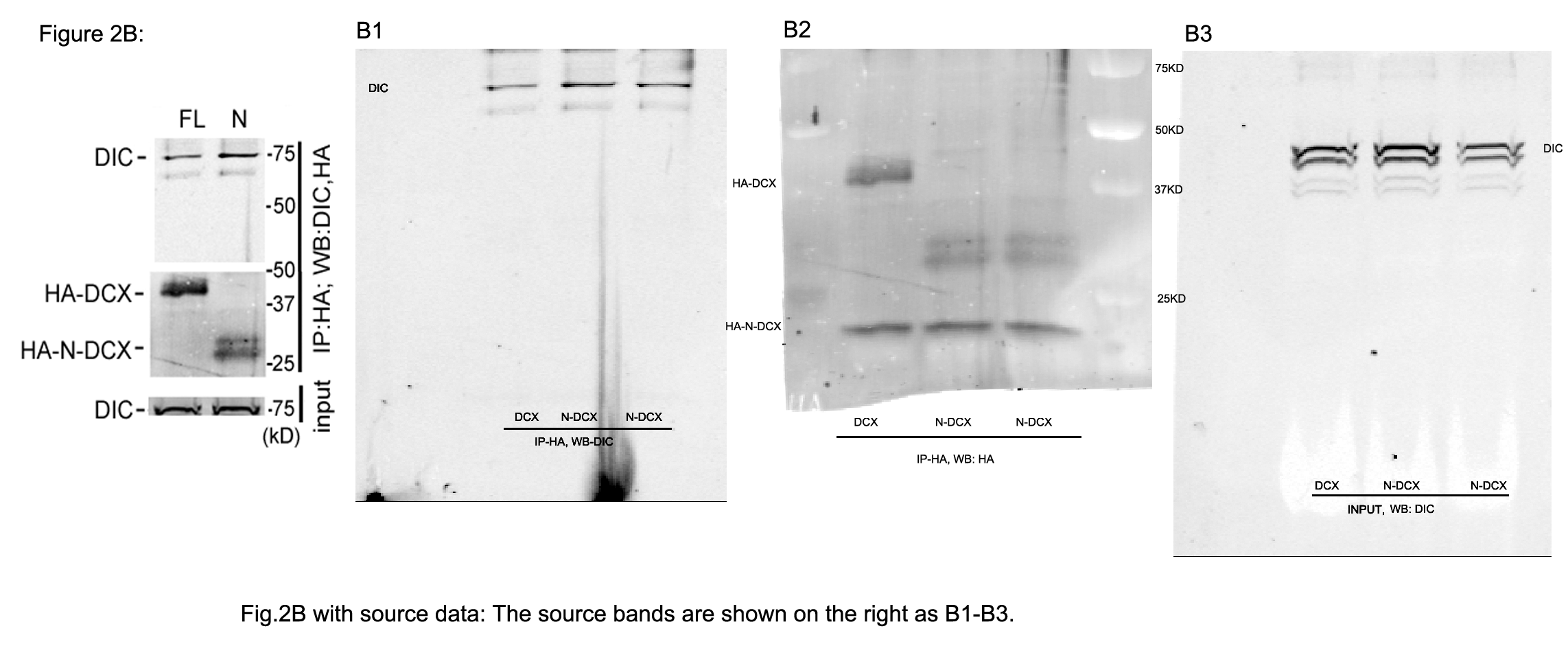

Supplement: Figure 2—source data 1. [file elife-82218-fig2-data1.zip › Fig2B-source data 4.tif]

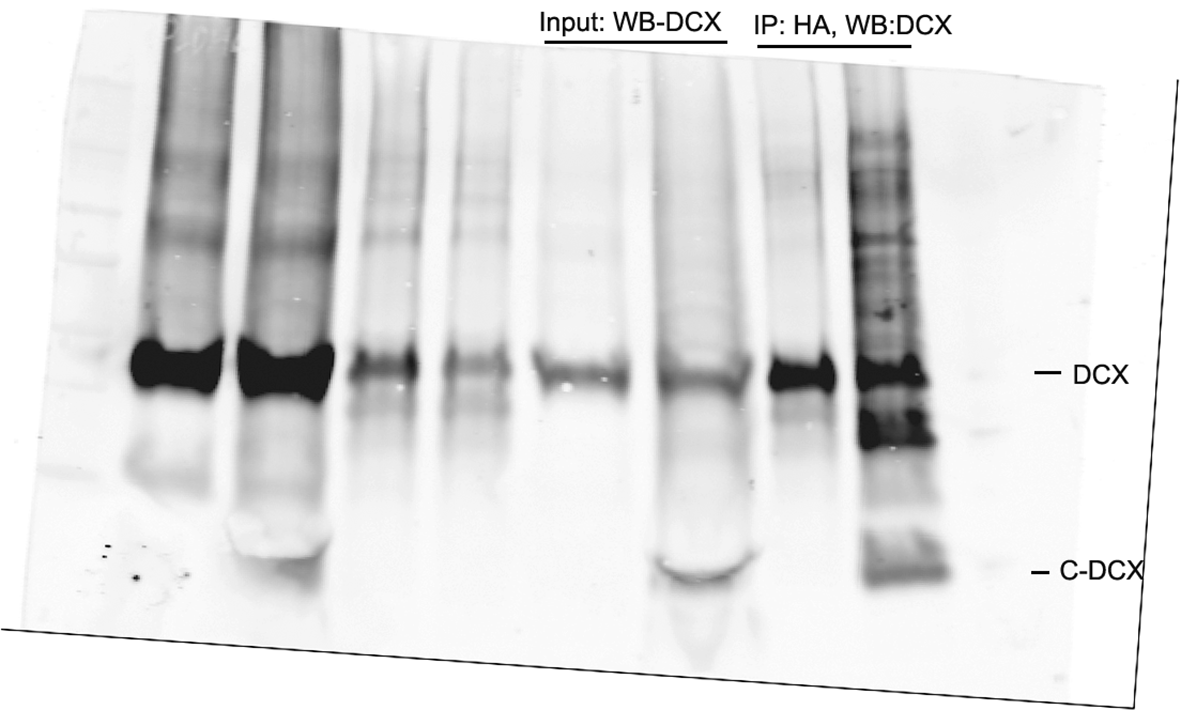

Supplement: Figure 2—source data 1. [file elife-82218-fig2-data1.zip › Fig2E-source data1.tif]

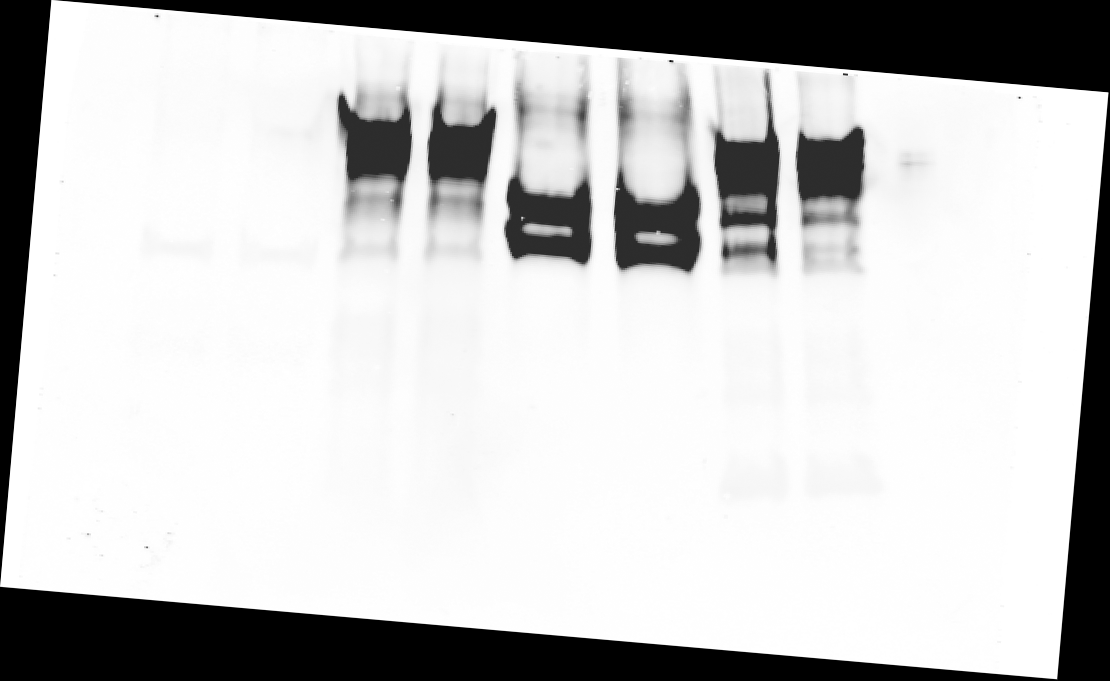

Supplement: Figure 2—source data 1. [file elife-82218-fig2-data1.zip › Fig2E-source data2.tif]

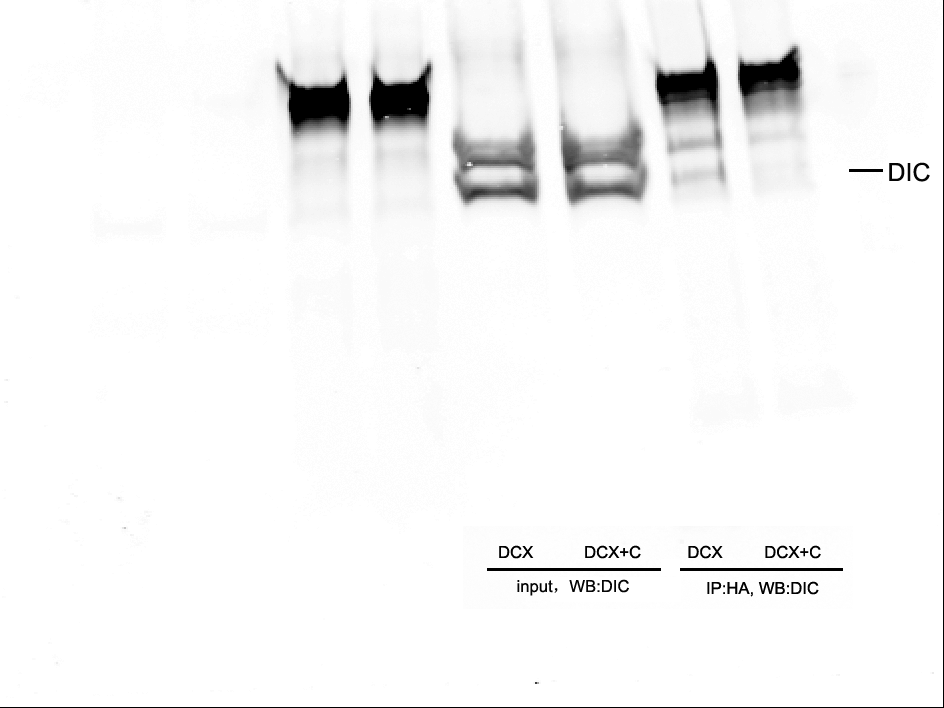

Supplement: Figure 2—source data 1. [file elife-82218-fig2-data1.zip › Fig2E-source data3.tif]

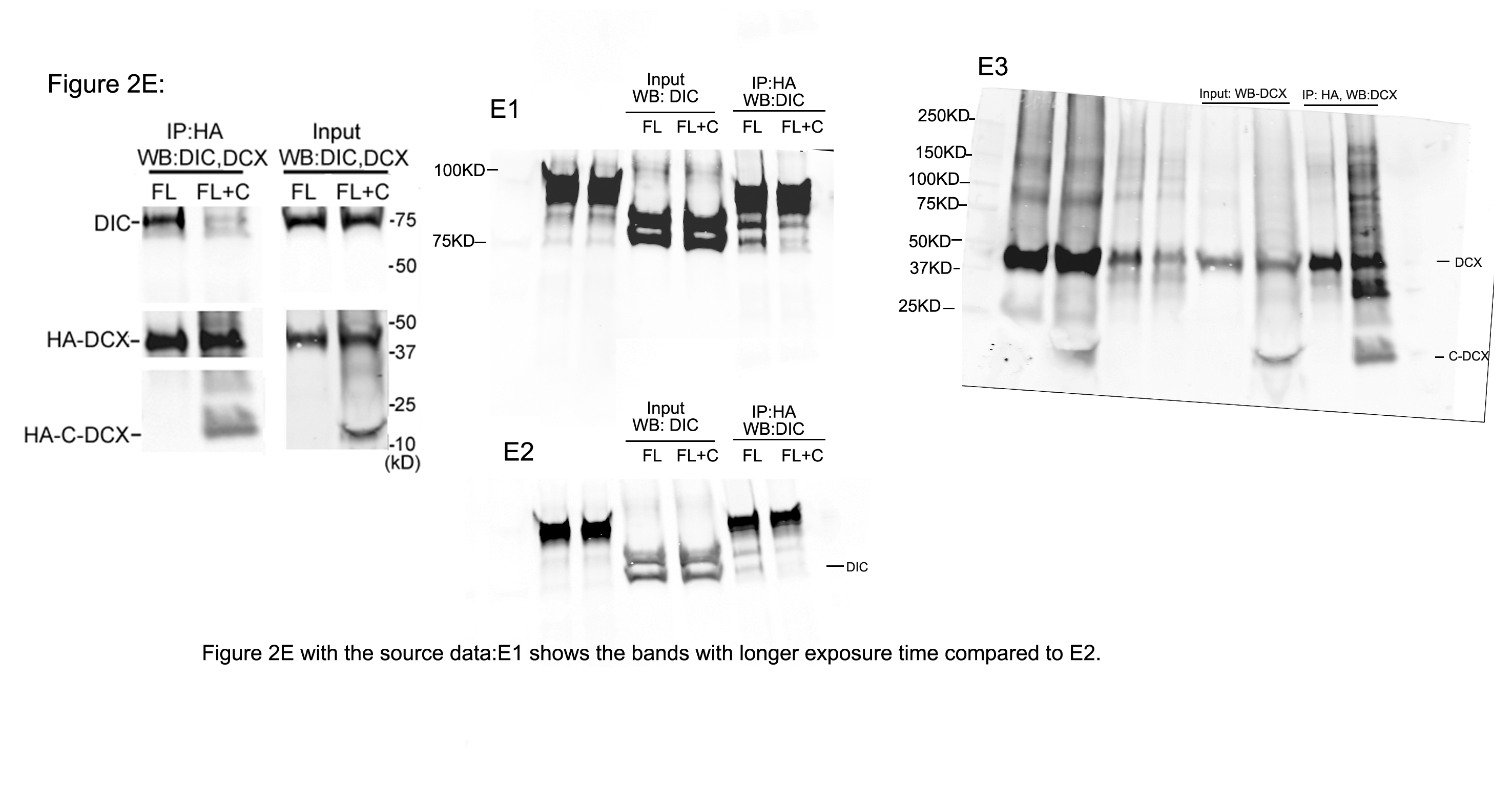

Supplement: Figure 2—source data 1. [file elife-82218-fig2-data1.zip › Fig2E-source data4.tif]

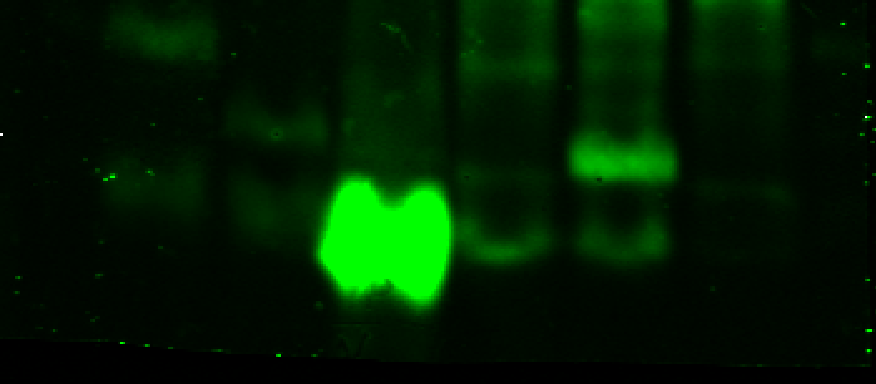

Supplement: Figure 2—figure supplement 1—source data 1. [file elife-82218-fig2-figsupp1-data1.zip › Fig2-figure supplement 1A source data 1.tif]

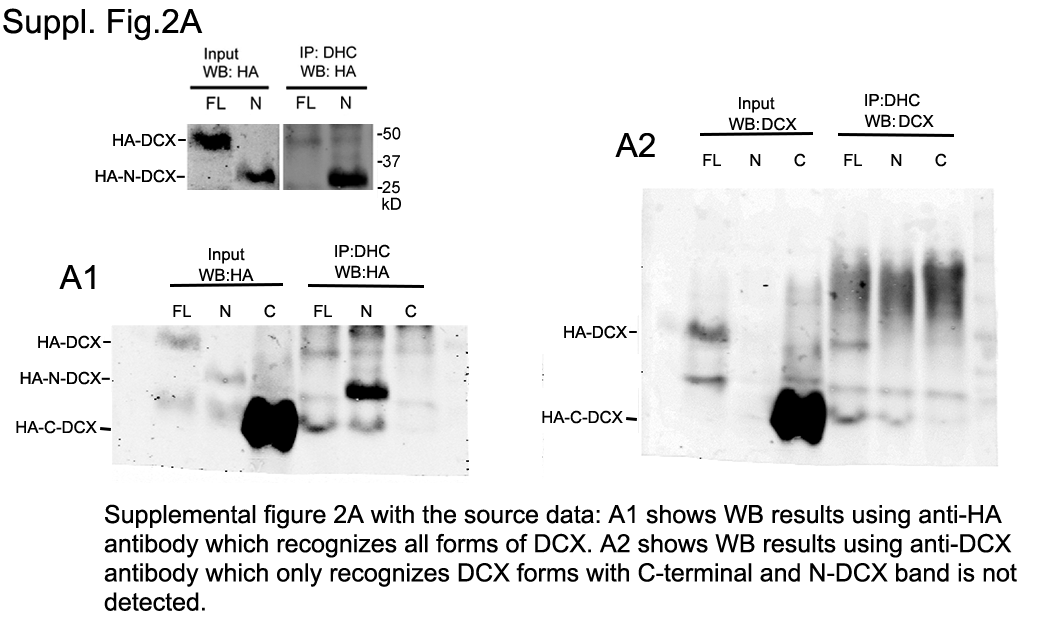

Supplement: Figure 2—figure supplement 1—source data 1. [file elife-82218-fig2-figsupp1-data1.zip › Fig2-figure supplement 1A with source data.tif]

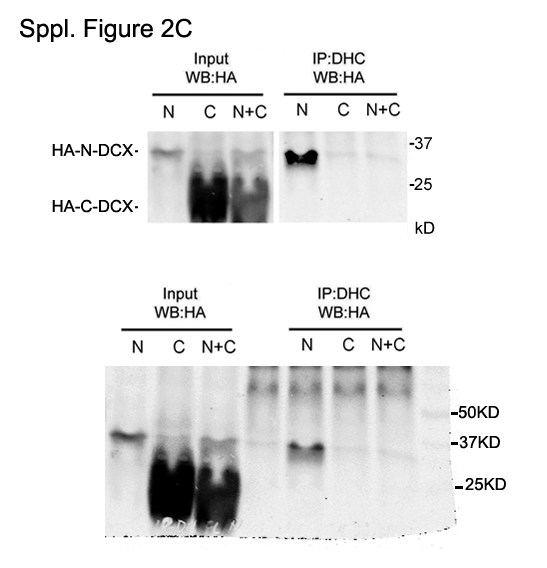

Supplement: Figure 2—figure supplement 1—source data 1. [file elife-82218-fig2-figsupp1-data1.zip › Fig2-figure supplement 1C with source data.tif]

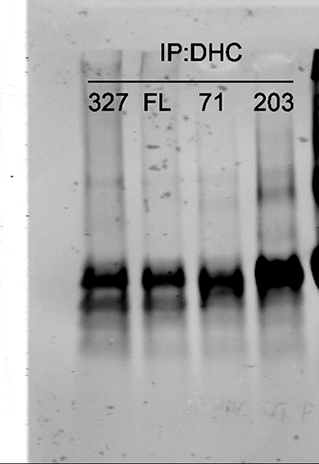

Supplement: Figure 2—figure supplement 1—source data 1. [file elife-82218-fig2-figsupp1-data1.zip › Fig2-figure supplement 1D source data 1.tif]

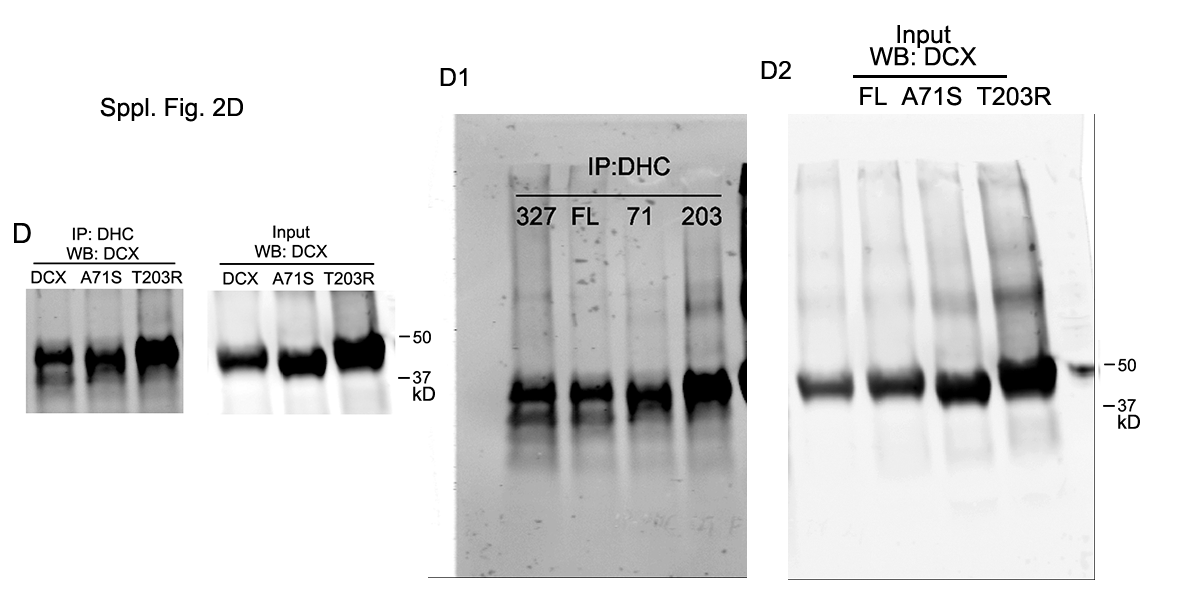

Supplement: Figure 2—figure supplement 1—source data 1. [file elife-82218-fig2-figsupp1-data1.zip › Fig2-figure supplement 1D with source data.tif]

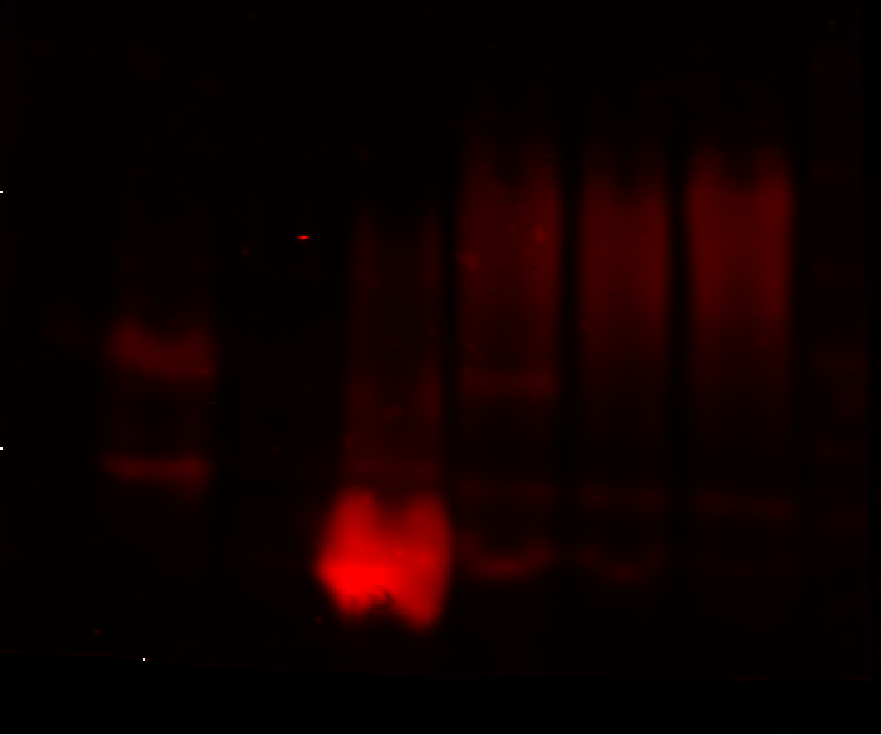

Supplement: Figure 2—figure supplement 1—source data 1. [file elife-82218-fig2-figsupp1-data1.zip › Fig2-figure supplement1A source data 2.tif]

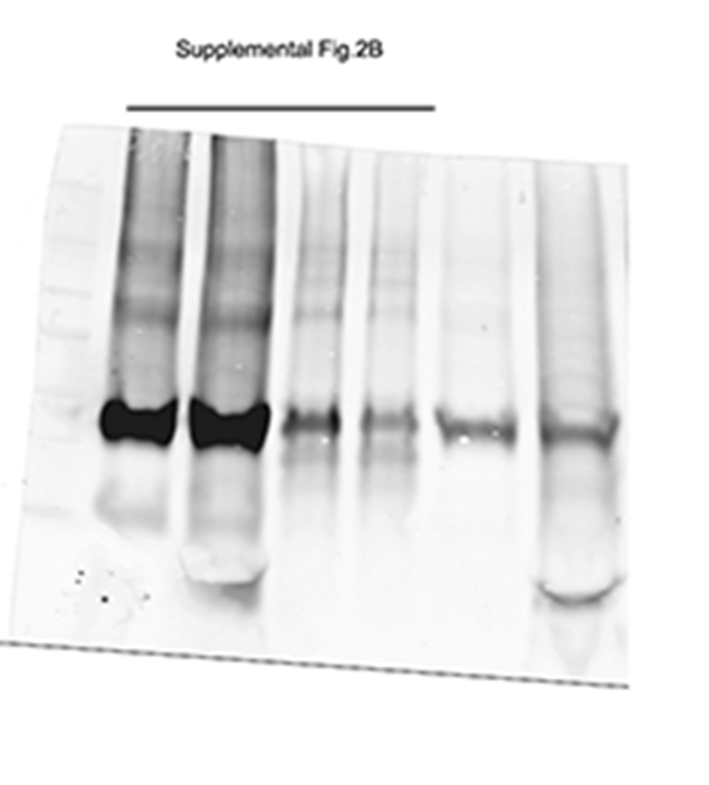

Supplement: Figure 2—figure supplement 1—source data 1. [file elife-82218-fig2-figsupp1-data1.zip › Fig2-figure supplement1B source data.tif]

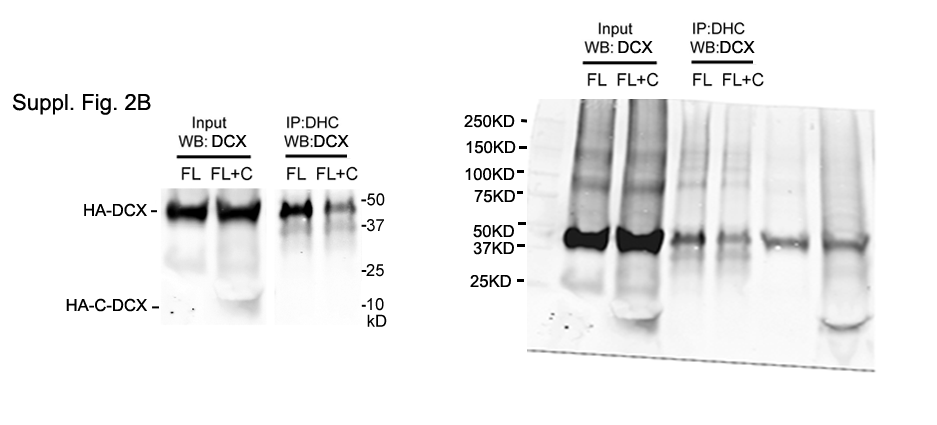

Supplement: Figure 2—figure supplement 1—source data 1. [file elife-82218-fig2-figsupp1-data1.zip › Fig2-figure supplement1B with source data.tif]

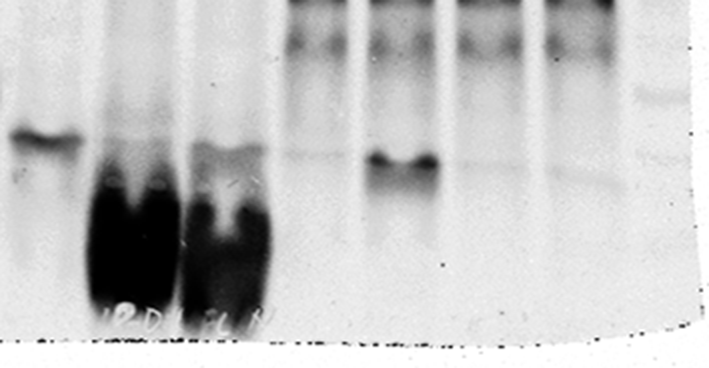

Supplement: Figure 2—figure supplement 1—source data 1. [file elife-82218-fig2-figsupp1-data1.zip › Fig2-figure supplement1C source data 1.tif]

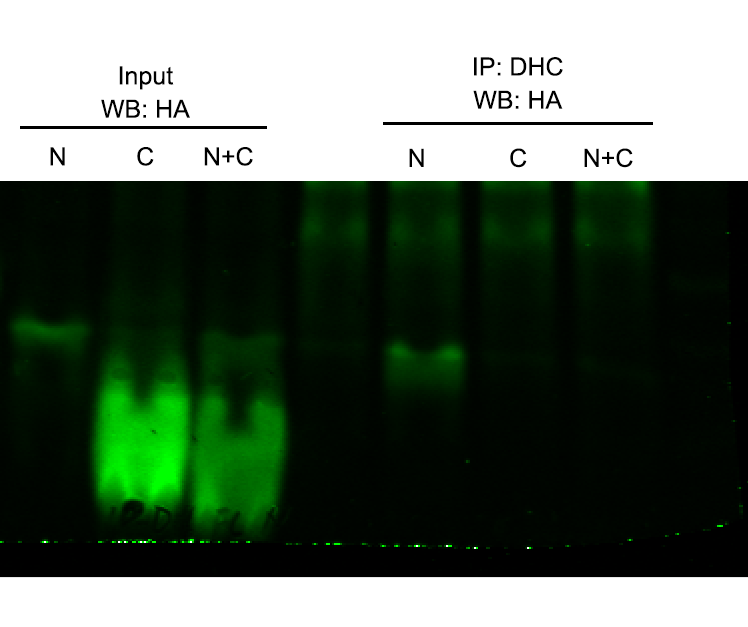

Supplement: Figure 2—figure supplement 1—source data 1. [file elife-82218-fig2-figsupp1-data1.zip › Fig2-figure supplement1C source data 2.tif]

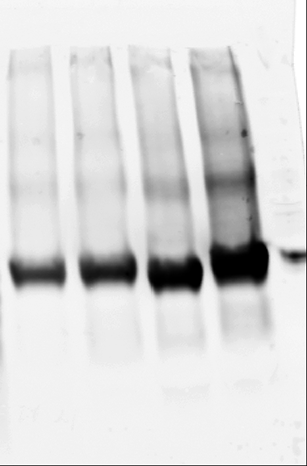

Supplement: Figure 2—figure supplement 1—source data 1. [file elife-82218-fig2-figsupp1-data1.zip › Fig2-figure supplement1D-source data 2.tif]

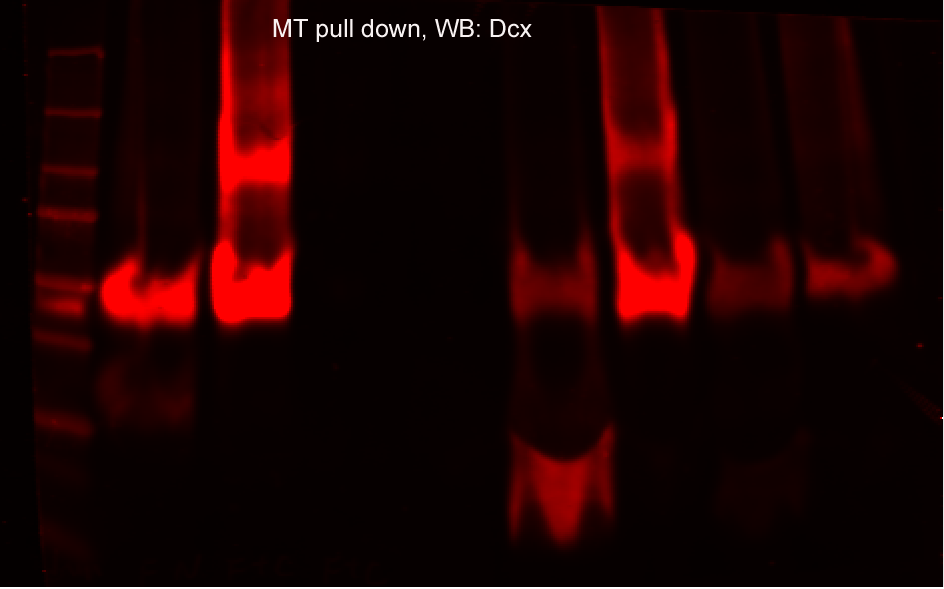

Supplement: Figure 3—source data 1. [file elife-82218-fig3-data1.zip › Figure 3B-source data 1.tif]

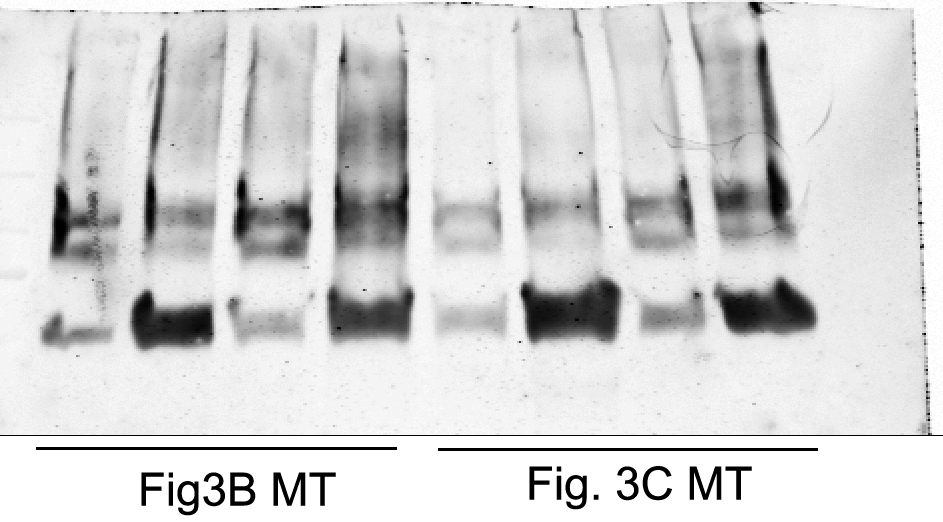

Supplement: Figure 3—source data 1. [file elife-82218-fig3-data1.zip › Figure 3B-source data 2.tif]

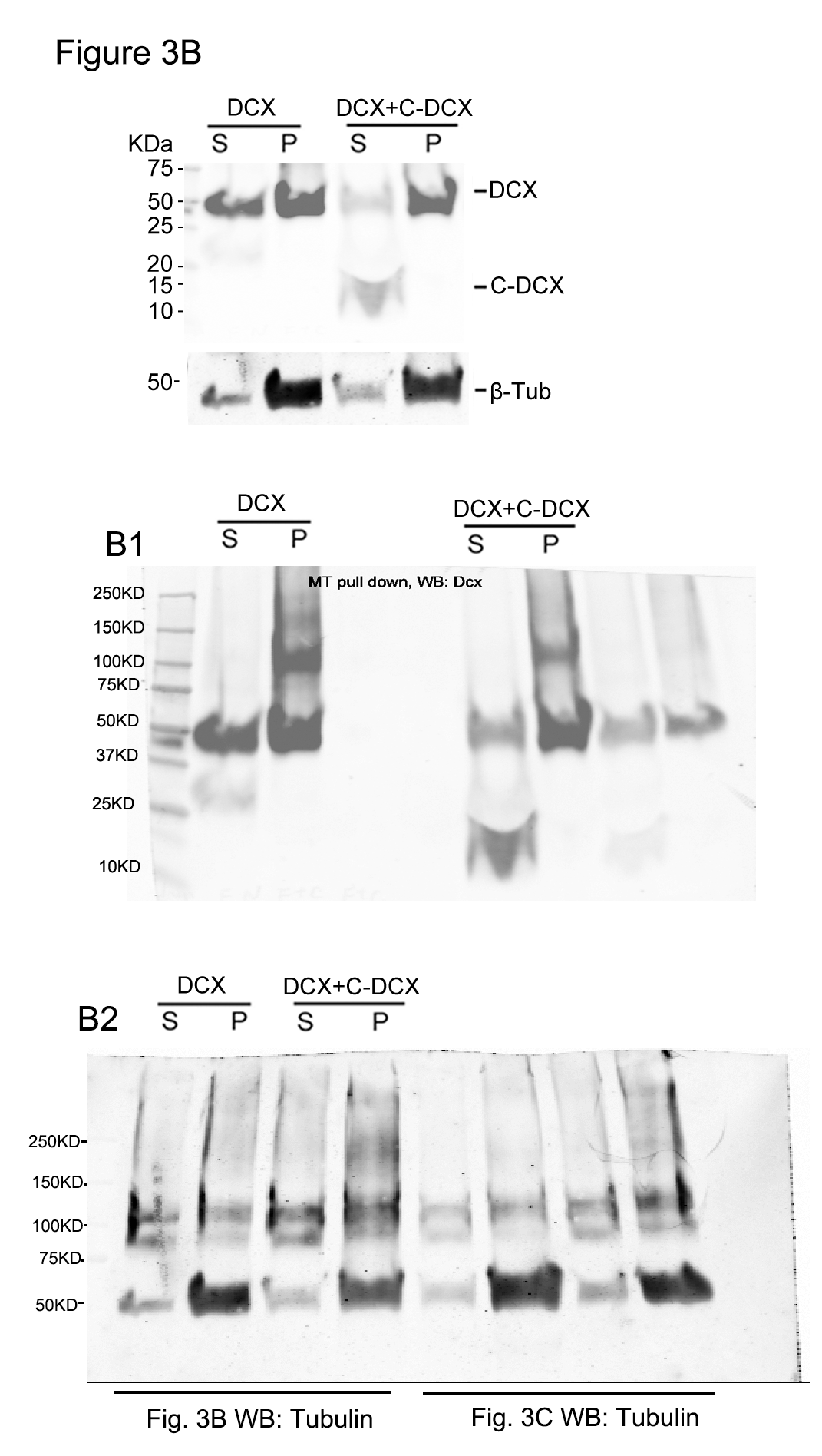

Supplement: Figure 3—source data 1. [file elife-82218-fig3-data1.zip › Figure 3B-source data 3.tif]

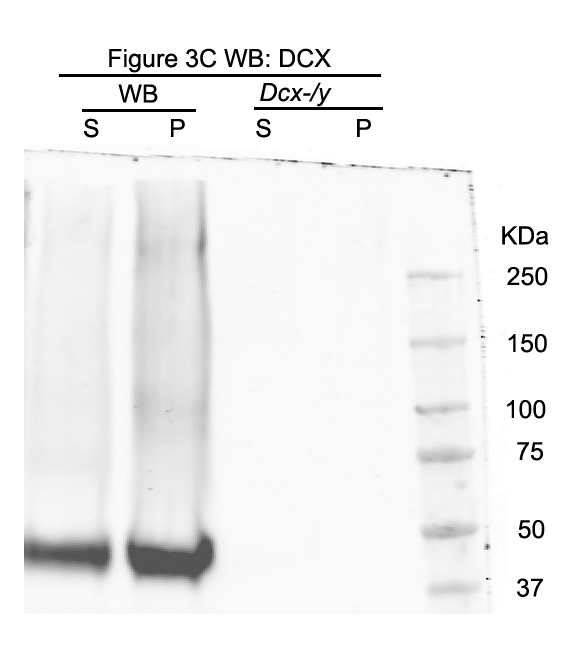

Supplement: Figure 3—source data 1. [file elife-82218-fig3-data1.zip › Figure 3C-source data 1.tif]

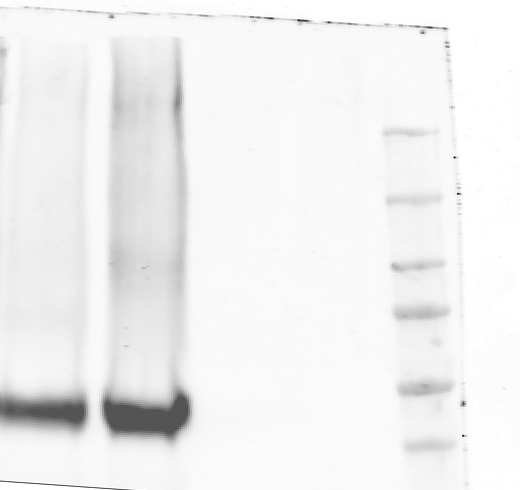

Supplement: Figure 3—source data 1. [file elife-82218-fig3-data1.zip › Figure 3C-source data 2.tif]

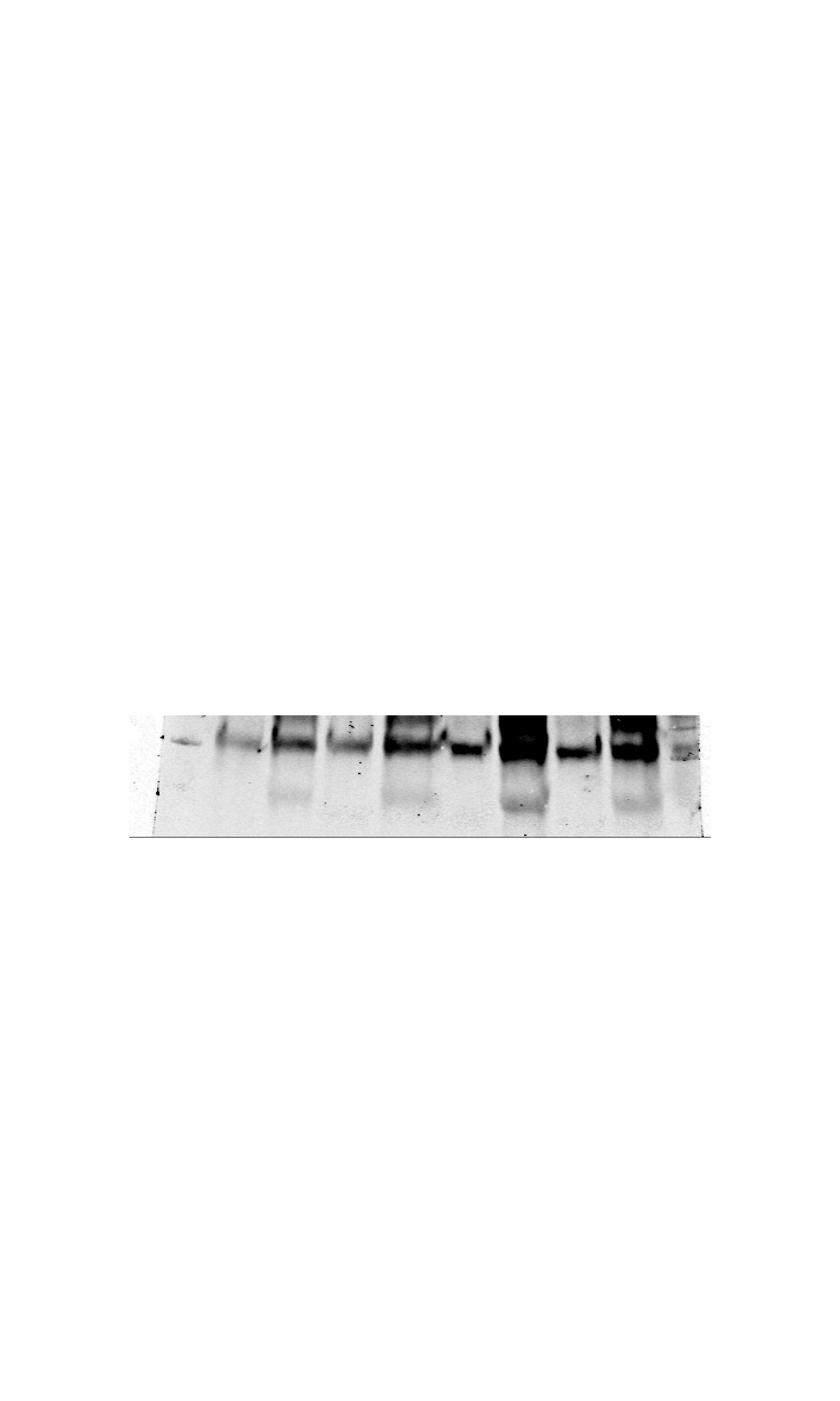

Supplement: Figure 3—source data 1. [file elife-82218-fig3-data1.zip › Figure 3C-source data 3.tif]

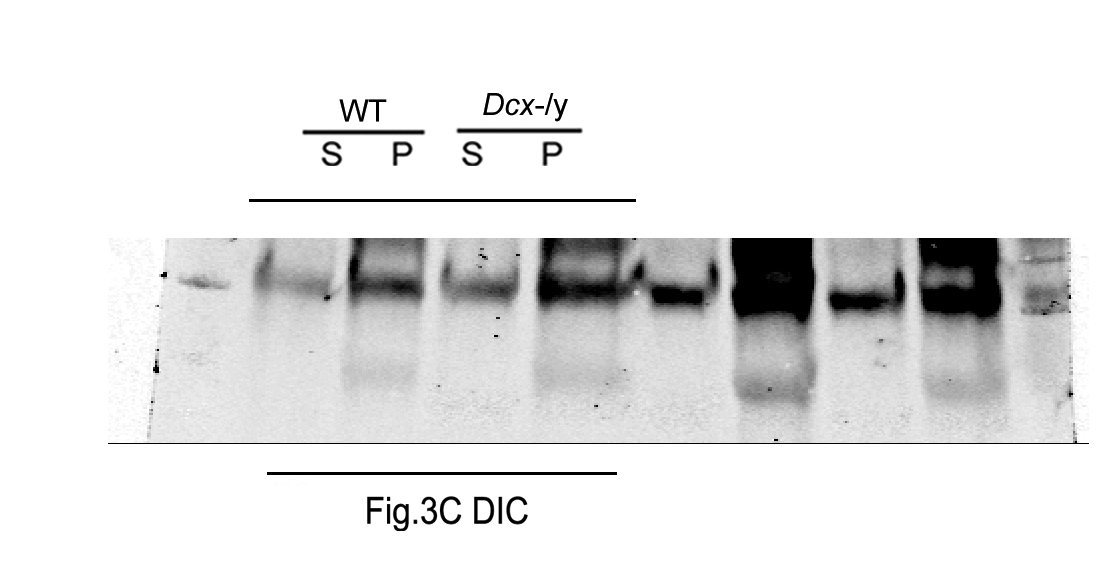

Supplement: Figure 3—source data 1. [file elife-82218-fig3-data1.zip › Figure 3C-source data 4.tif]

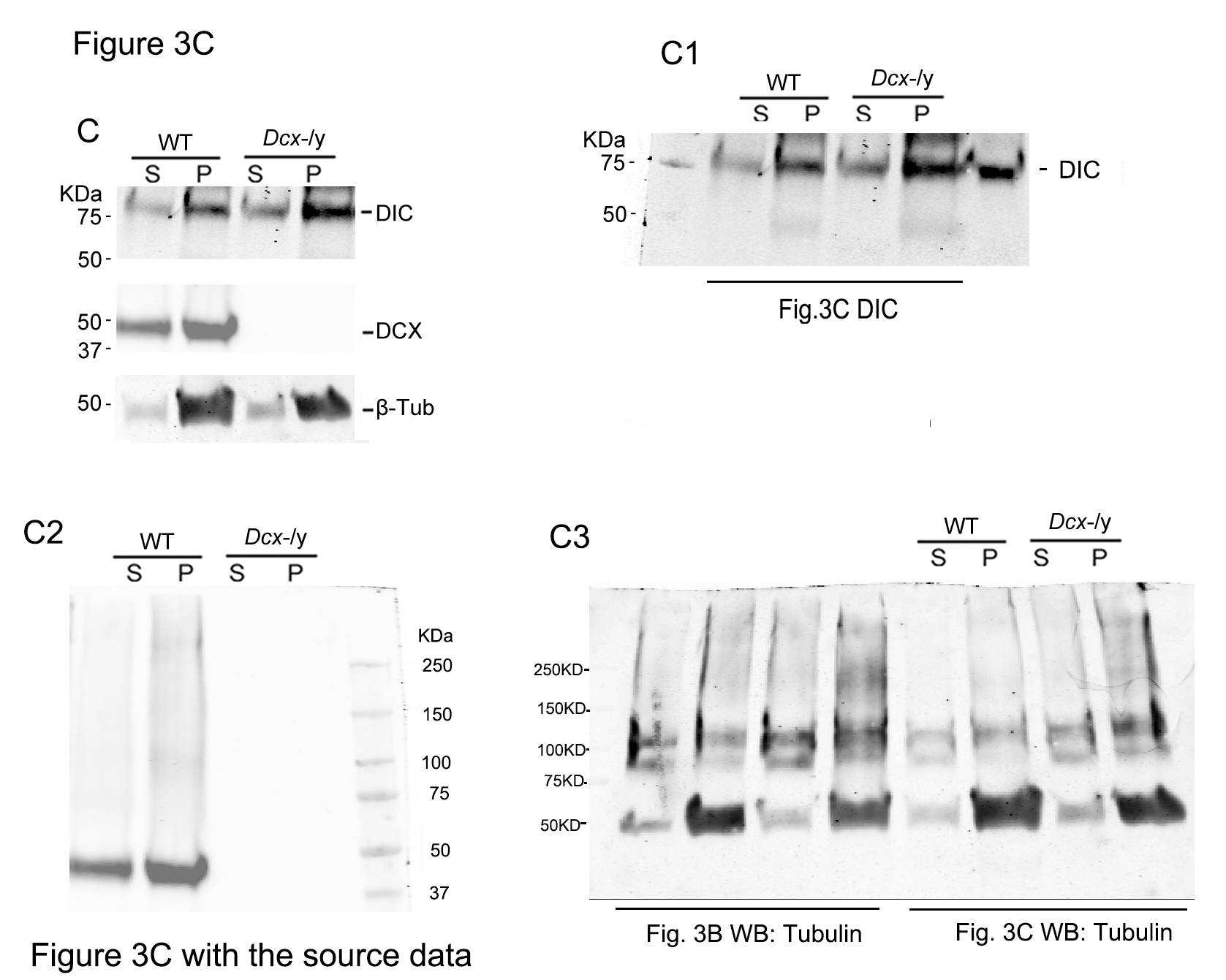

Supplement: Figure 3—source data 1. [file elife-82218-fig3-data1.zip › Figure 3C-source data 5.tif]

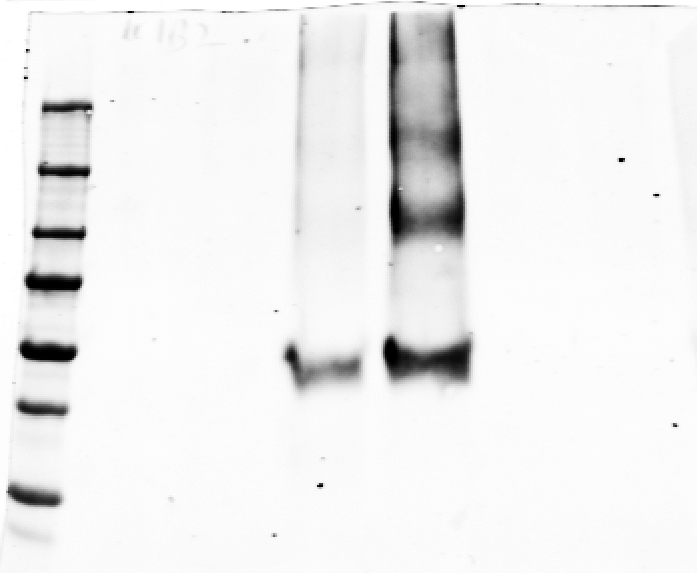

Supplement: Figure 3—source data 1. [file elife-82218-fig3-data1.zip › Figure 3D-source data 1.tif]

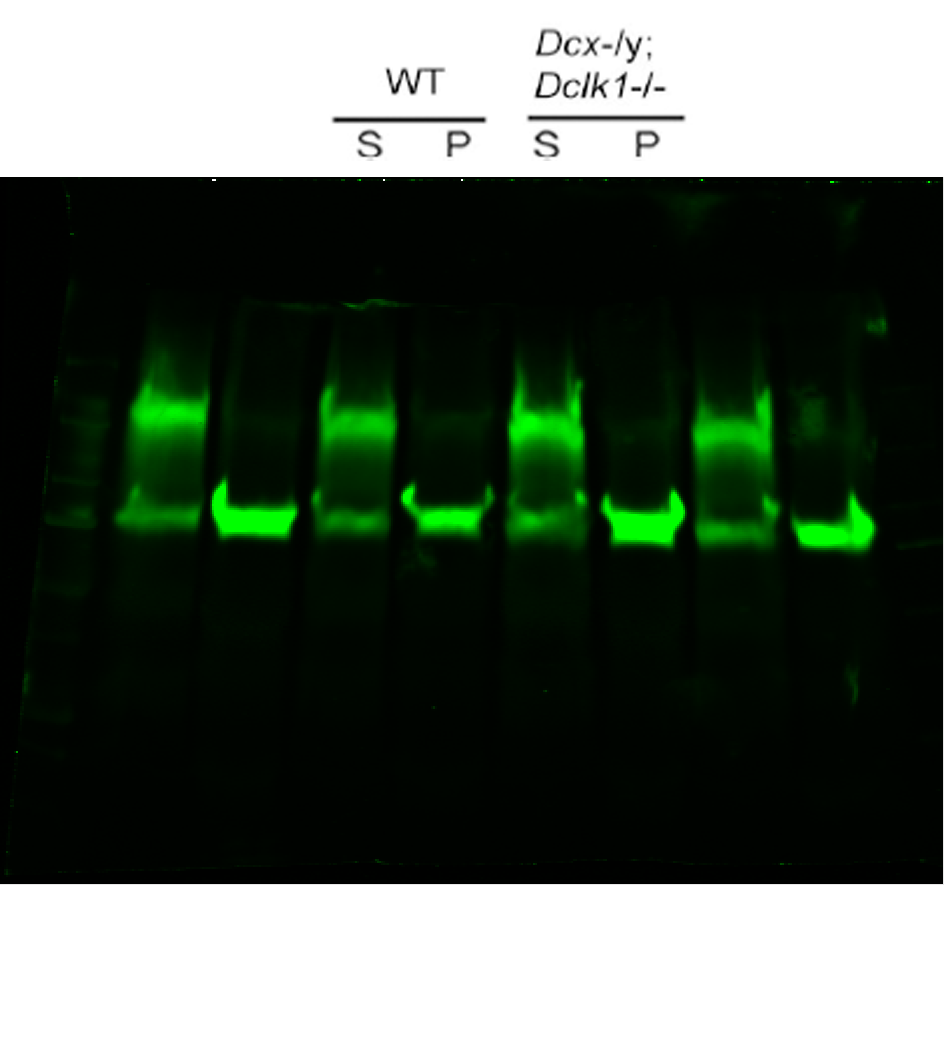

Supplement: Figure 3—source data 1. [file elife-82218-fig3-data1.zip › Figure 3D-source data 2.tif]

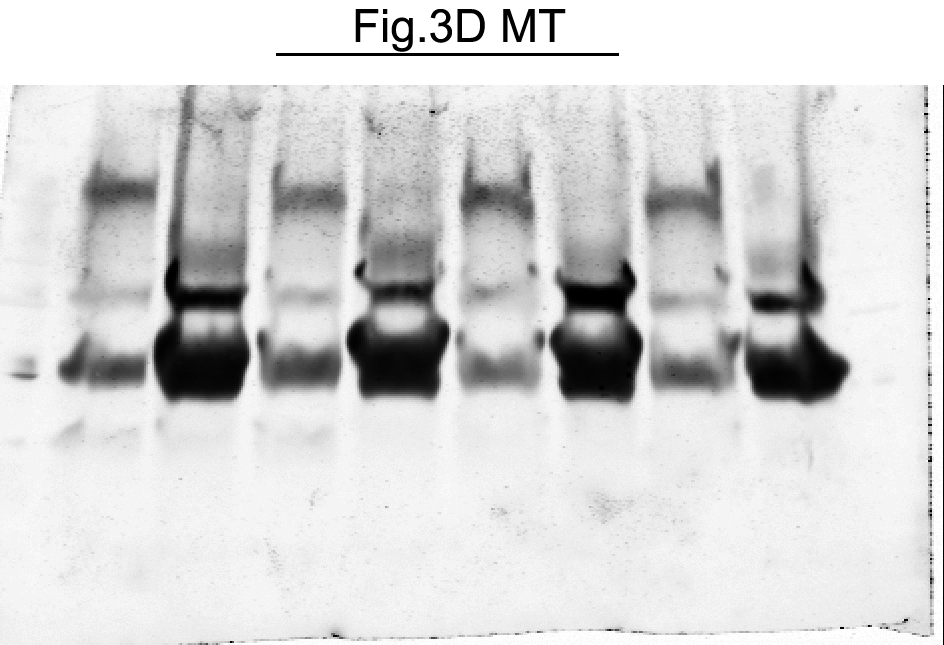

Supplement: Figure 3—source data 1. [file elife-82218-fig3-data1.zip › Figure 3D-source data 3.tif]

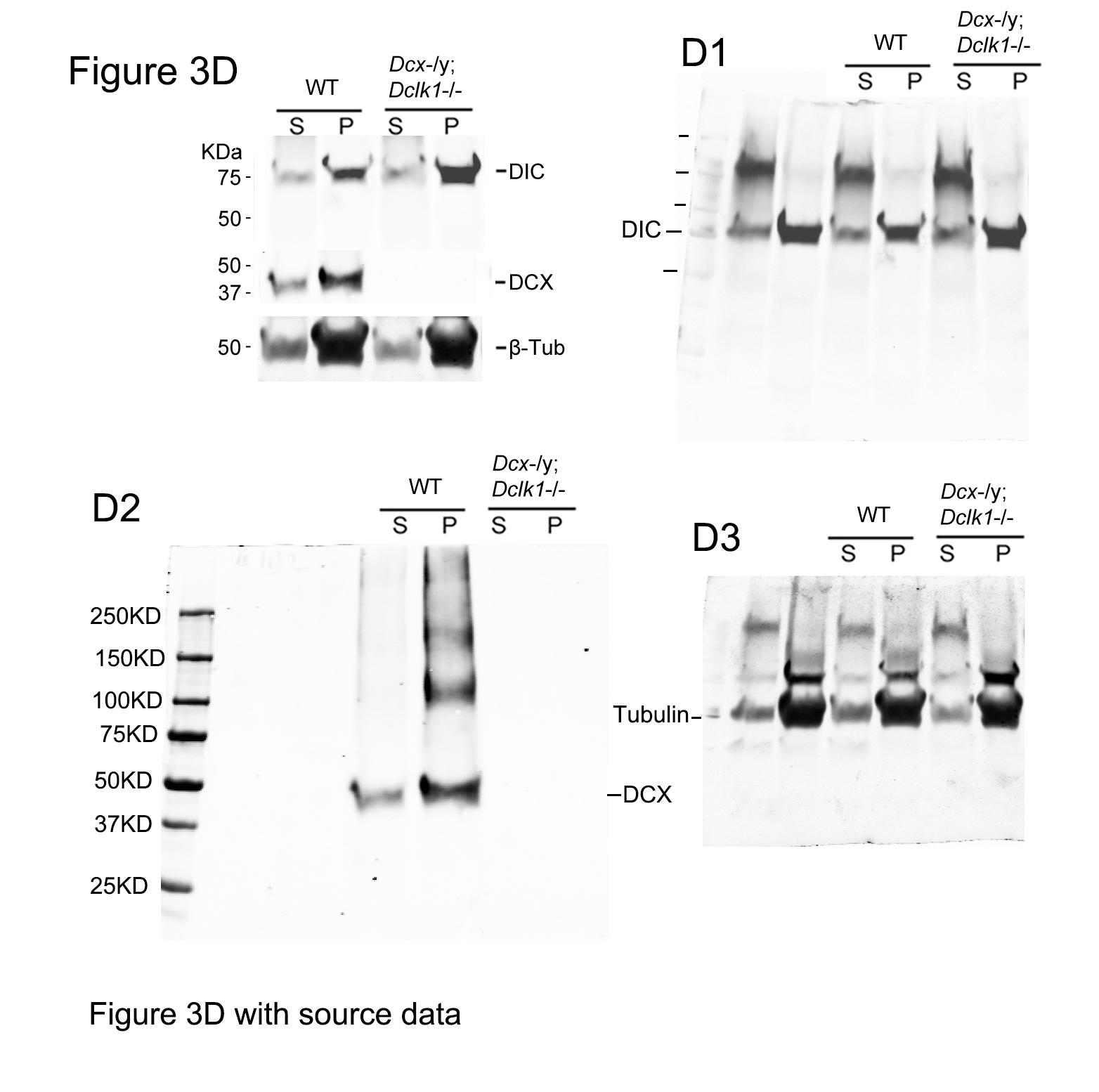

Supplement: Figure 3—source data 1. [file elife-82218-fig3-data1.zip › Figure 3D-source data 4.tif]

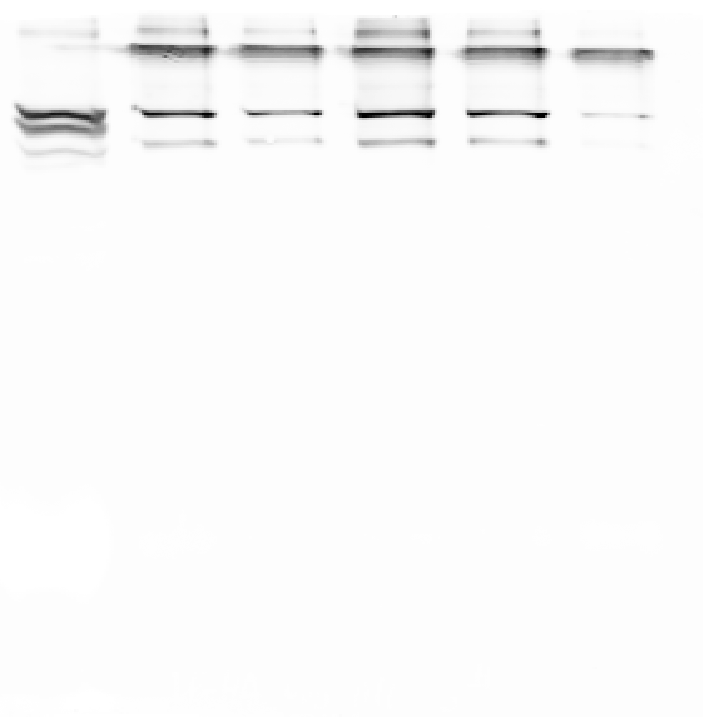

Supplement: Figure 4—source data 1. [file elife-82218-fig4-data1.zip › Figure 4-source data 1.tif]

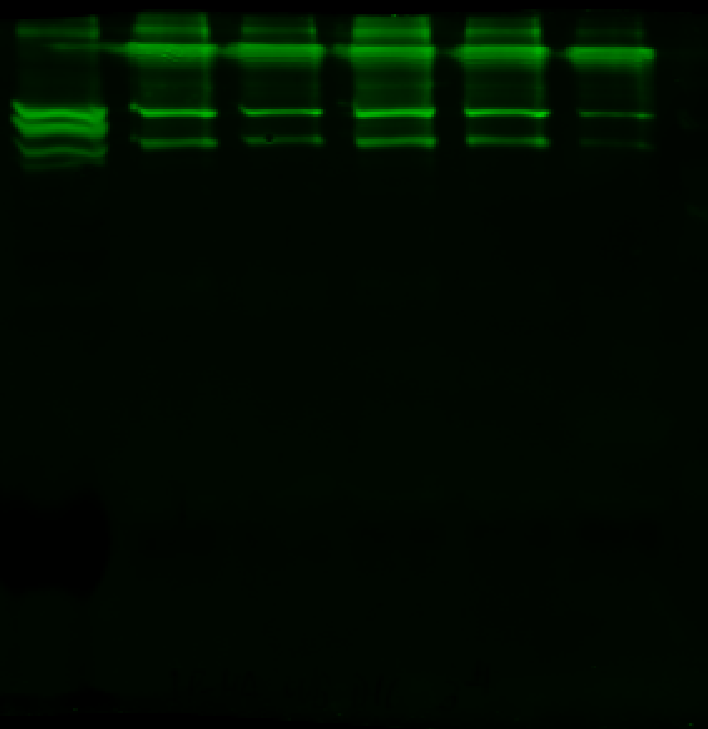

Supplement: Figure 4—source data 1. [file elife-82218-fig4-data1.zip › Figure 4-source data 2.tif]

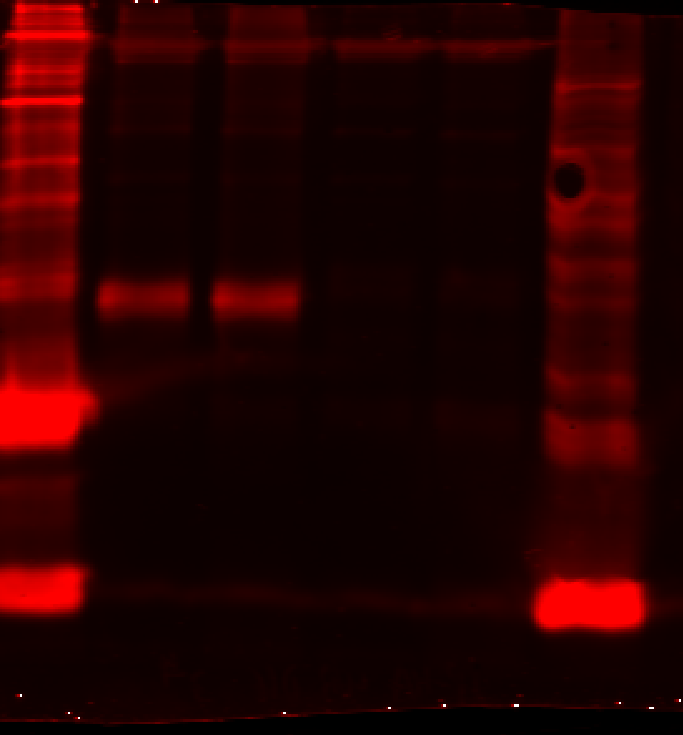

Supplement: Figure 4—source data 1. [file elife-82218-fig4-data1.zip › Figure 4-source data 3.tif]

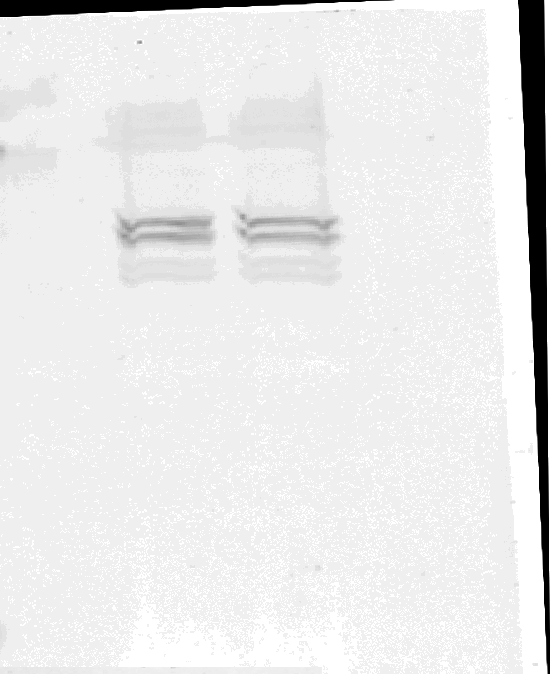

Supplement: Figure 4—source data 1. [file elife-82218-fig4-data1.zip › Figure 4-source data 4.tif]

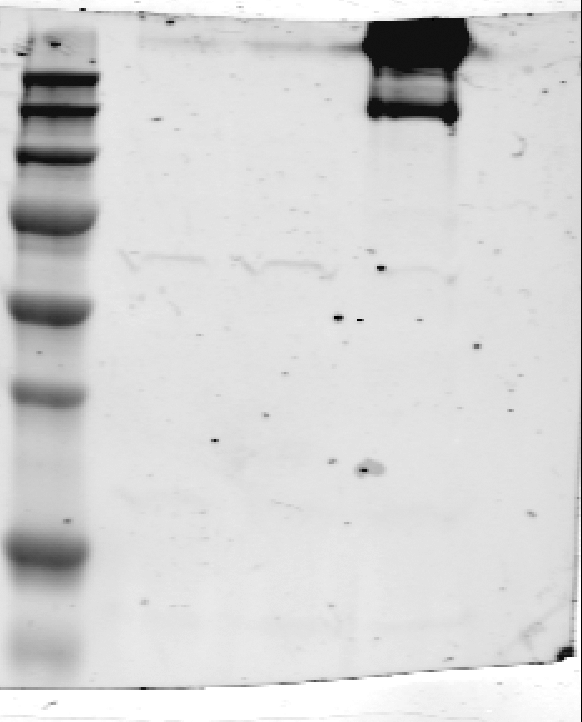

Supplement: Figure 4—source data 1. [file elife-82218-fig4-data1.zip › Figure 4-source data 5.tif]

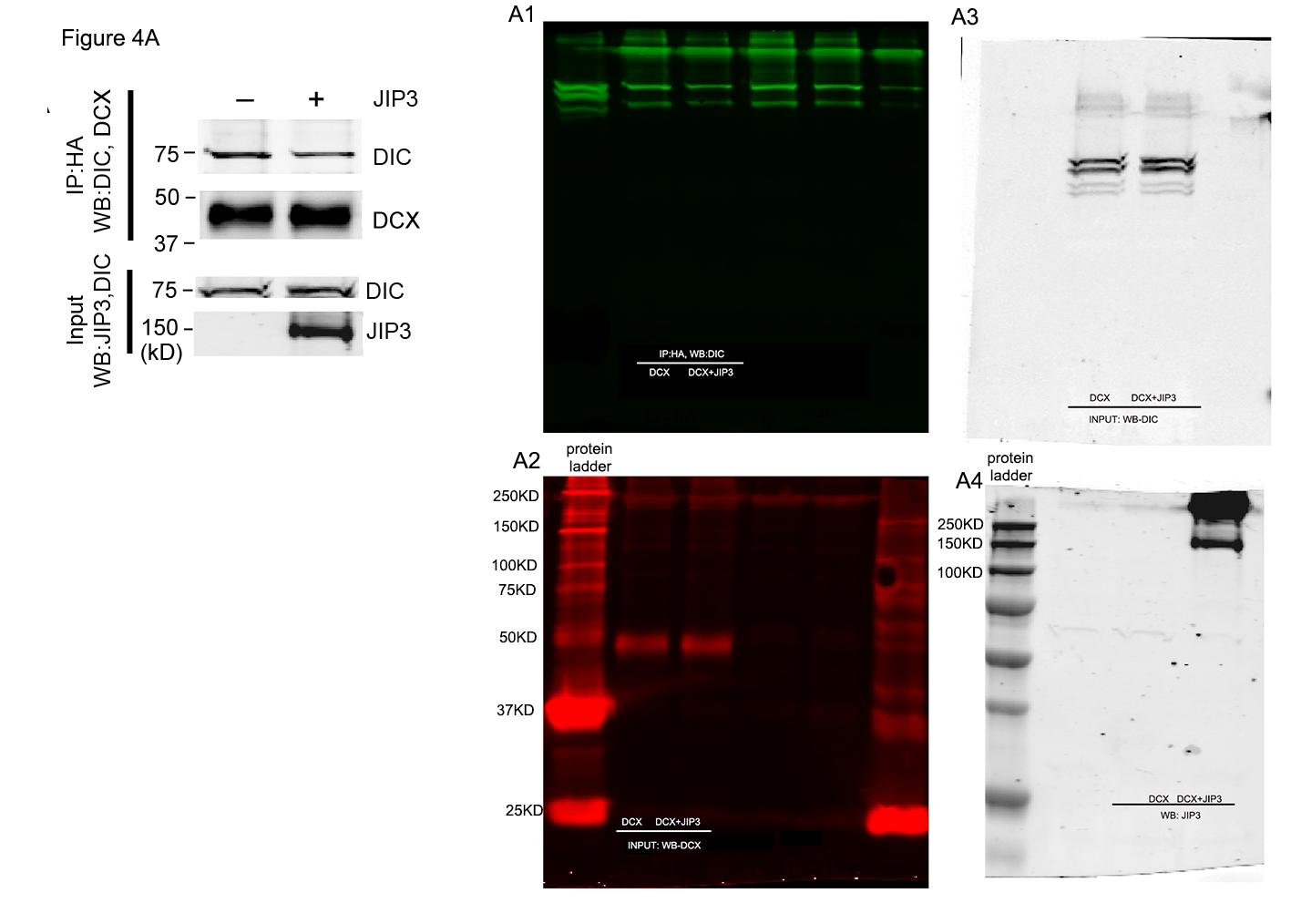

Supplement: Figure 4—source data 1. [file elife-82218-fig4-data1.zip › Figure 4-source data 6.tif]
